# Assessment of measurement properties of the Brazilian-Portuguese version of the low back activity confidence scale (LoBACS) in patients with chronic low back pain

**DOI:** 10.1371/journal.pone.0239332

**Published:** 2020-09-22

**Authors:** Josilainne Marcelino Dias, Mariana Felipe Silva, Laís Faganello Dela Bela, Alexandre Roberto Marcondes Pelegrinelli, Mabel Micheline Olkoski, Leandro Caetano Guenka, Kimiko Yamada, Jefferson Rosa Cardoso

**Affiliations:** 1 Medicine School, Universidade Estadual de Mato Grosso do Sul, Campo Grande, Mato Grosso do Sul, Brazil; 2 Laboratory of Biomechanics and Clinical Epidemiology, PAIFIT Research Group, Universidade Estadual de Londrina, Londrina, Parana, Brazil; 3 Positive University, Curitiba, Parana, Brazil; 4 Laboratory of Applied Biomechanics, Universidade Estadual de Londrina, Parana, Brazil; 5 Department of Forest Engineering, Agroveterinary Sciences Center, Universidade do Estado de Santa Catarina, Lages, Santa Catarina, Brazil; 6 Division of Biokinesiology and Physical Therapy at the Ostrow School of Dentistry, University of Southern California, Los Angeles, California, United States of America; Western University, CANADA

## Abstract

The Low Back Activity Confidence Scale (LoBACS) assesses the self-efficacy to perform activities in individuals with chronic low back pain (CLBP). As self-efficacy appears to directly influence the patient’s functional capacity and prognosis, it is important to develop a scale that evaluates this attribute to guide treatment strategy and monitor the clinical course of patients. This study aimed to evaluate the reliability, construct validity, and responsiveness of the Brazilian version of the LoBACS. The scale was applied to 112 male and female patients (age, 18–65 years) with specific and nonspecific CLBP. For evaluating the interobserver reliability, the scale was applied twice on the first evaluation day by two trained evaluators (A and B). Within 48–72 h of the first evaluation, assessor A reapplied the scale to evaluate intraobserver reliability (test–retest), which was analyzed by intraclass correlation coefficient (ICC). The first LoBACS applied in the baseline evaluation was also used to assess the construct validity of the scale by factor analysis. For responsiveness, the scale was applied 5 times at 2-week intervals and the change in scores was analyzed by the repeated measures ANOVA. Although factor analysis indicated three subscales, they did not present acceptable values of convergent and divergent validity. Reliability ranged from good to excellent, with ICC values of .90 (95% CI, .84; .93) and .85 (95% CI, .77; .91) for inter- and intraobserver variability for total score. Moreover, the total score was responsive in all comparisons, with no floor or ceiling effects. Thus, only the total score of the Brazilian version of LoBACS proved to be reliable, valid, and responsive.

## Introduction

In Brazil, chronic low back pain (CLBP) affects nearly 18.5% (95% confidence interval [CI], 17.8–19.1) of individuals >18 years of age, with 16.4% of them presenting with severe disability [1]. The global economic burden from the disease is highly significant, with about $200 billion annual spend [2].

CLBP is attributed to intricate biopsychosocial mechanisms and multiple risk factors [3, 4]. Several studies have evaluated the relationship between CLBP and self-efficacy (individual’s confidence in their ability to perform a task) and found that cognitive tests, such as assessment of functional self-efficacy, have greater explanatory power than specific pain-related assessments [5–9]. In addition, associations were found between low self-efficacy and higher disability rates [7, 10]. Patients with CLBP and low self-efficacy are about 113% more likely to present with disability than patients with high self-efficacy [11]. Moreover, psychological factors have been established as predictors of chronicity in patients with low back pain (LBP) [12].

Recently, some studies have evaluated self-efficacy as a treatment method for patients with LBP [13–15]. Khodadad *et al*. compared the effectiveness of cognitive functional approach and lumbar stabilization and found no difference between the methods [14]. Both methods were able to improve pain and movement control [14]. Ferrari *et al*. evaluated the effect of physical therapy centered on cognitive and behavioral principles on patients with lumbar spondylolisthesis and CLBP. Their results indicated an improvement in lumbar function, pain self-efficacy, and clinical tests [15].

Self-efficacy seems to be an important outcome in evaluating patients with CLBP as it can predict disability [5–11], adherence to treatment [16], and the likelihood of chronicity [12]. Therefore, it is important to develop a self-efficacy evaluation questionnaire that can assist in providing better physical and biopsychosocial treatment for these patients.

In Brazil, this outcome has been used to assess different health conditions and for specific populations. However, there are no questionnaires formulated specifically for patients with LBP or for evaluating the self-regulation domain [17–20]. It is also well known that questionnaires formulated in a foreign language are not suitable for use in all populations because of differences in beliefs, definitions, and lifestyles in different cultures [21, 22]. The Low Back Activity Confidence Scale (LoBACS) was translated into and cross-culturally adapted to Brazilian–Portuguese [23]. The changes made during this adaptation require evaluation of measurement properties to verify both internal and external validity. The present study aimed to evaluate the reliability, construct validity, and responsiveness of the Brazilian version of LoBACS in patients with CLBP.

## Materials and methods

This is a measurement property evaluation study [22, 24]. All changes made during the process of evaluation of LoBACS into Portuguese were authorized by the authors of the original scale. Additionally, this study was approved by the Universidade Estadual de Londrina–Institutional Review Board #17918313.4.0000.5231.

### Sample

A consecutive sampling method was used. The sample was selected through television and radio advertisements. Patients were assessed for inclusion criteria by two physical therapists trained for evaluation. A medical diagnosis of CLBP (more than 12 weeks) was requested [25]. Inclusion criteria were individuals with specific and nonspecific CLBP, of both genders, aged between 18 and 65 years, and referred to physical therapy. Patients were excluded if they had fibromyalgia, ankylosing spondylitis, or rheumatoid arthritis; were immediately postoperative for lumbar spine or lower limbs or refused to sign the informed consent.

The sample size calculation was based on the goodness of fit test through the G*Power program and accounted for a 0.4 effect size, an error of 5%, a power (1-β) of 95%, and two degrees of freedom. The sample size was 110 patients, considering 10% of dropouts.

### Instruments

**LoBACS** is a tool that measures the self-efficacy of patients with LBP, can be self-applicative, and consists of 15 items divided into three subscales: Functional self-efficacy (FnSE) composed of seven items–related to the confidence to perform functional activities; Self-regulatory self-efficacy (Self-RegSE) consisting of three items, which assesses the confidence to handle and control problems related to the lumbar spine, and Exercise self-efficacy (ExSE), consisting of five items related to the confidence and proactivity to perform physical exercises [26, 27]. This instrument consists of a Likert scale of 11 points, ranging from 0% (no confidence) to 100% (complete confidence) with increments of 10% for each point. Scale scoring can be calculated globally (e.g., the average score of all items), or domain by domain (e.g., the average score of items 1–7 for FnSE subscale), and the total score may range from 0 to 100, where the higher the score, the better the self-efficacy [26]. When a questionnaire item is not answered, it should be excluded from the average in the final calculation.

The process to create the Brazilian-Portuguese version of the LoBACS was performed according to the already established protocol [28]: *Preparation and Forward translation*—four professionals who are native speakers of Portuguese with English as a second language conducted an independent translation from English to Portuguese (T1; T2; T3; T4); *Reconciliation*—the Portuguese versions were analyzed and discussed, with discrepancies documented in summary, to produce a single version. At this stage, some unit of measurement amendments were performed; for example, the use of kg instead of lb; *Back translation*—the single version in Portuguese was submitted to a new translation into English by three translators (BT1, BT2, and BT3), who were native English speakers with a second language of Portuguese; *Back translation review and Harmonization—*back translated versions were compared with the original scale to obtain a pretesting version (PTV) in Portuguese [23].

This PTV was applied to a selected sample of 37 individuals. They were questioned about their understanding, doubts, and suggestions during the *Cross-cultural adaptation stage or Cognitive debriefing*. After this, a *Review of cognitive debriefing results* was conducted, and if appropriate, amendments were made. Modifications were as follows: Question 1 –change the word “box” to “object”; Question 2 –change the word “phone book” to “book”; Question 3 –change the word “carpet” to “rough surface (such as carpet)”; Question 4 –the word “plane” was removed; and Question 5 –the number of steps in a flight of stairs was included. After this stage, *Proofreading and Final report* of the final Brazilian-Portuguese version of the scale was obtained (S1 File) [23].

### Procedures

The reliability of the Brazilian version of LoBACS was evaluated by applying it twice on the first evaluation day: two protocol-blinded and trained evaluators (**A** and **B**) applied the LoBACS with an interval of two hours (inter-observer reliability). After the first assessment, between 48 and 72 hours, assessor **A** reapplied the scale to evaluate intra-observer reliability (test-retest). The first LoBACS-Brasil applied in the baseline evaluation was also used to evaluate the construct validity of the scale by factor analysis.

To assess responsiveness, the scale was applied five times, over eight weeks of treatment, with two-week intervals between each application, and the change in the score was analyzed. The sequence of treatment was showed in the S1 Table.

### Data analysis

Numerical variables were assessed for the distribution of normality by the Shapiro-Wilk test and are presented as medians (Md) and quartiles (25–75%). Categorical variables are presented as absolute and relative frequencies. For evaluation of intra- and inter-rater reliability, the intraclass correlation coefficient (ICC_3,1_) two-way mixed model, with 95% CI, which is based on one-way ANOVA, was used. The standard error of the measurement (SEM) was calculated using the formula = SD x √(1-CCI), where the SD was derived from the mean square of the residuals [29]. Reliability was considered poor when the value of the ICC was < .40, good when .40 ≤ ICC ≥ .75, and excellent for values >.75 [30].

Davenport *et al*. evaluated latent factors of the original scale through principal component analysis and found 3 factors [27]. In this study, the quality of the structure found was tested using confirmatory factor analysis (CFA) through the maximum likelihood estimation with robust standard errors, followed by a mean and variance adjusted test statistic (WLSMV). The model was adjusted until acceptable values were found in these fit tests: comparative fit index (CFI), Tucker-Lewis index (TLI), and root mean square error of approximation (RMSEA). For CFI to TLI the values were considered great when > .95, traditional when > .90, and sometimes permissible when > .80. For RMSEA the values were good when < .05, moderate between .05-.10 and bad when >.10 [31].

Both the reliability and validity of the scale structure were evaluated through the following tests: composite reliability (CR) (>.70), average variance extracted (AVE) (>.50), and maximum shared variance (MSV) (MSV < AVE) [32]. The systematic error of the data was analyzed by the common method bias through the common latent factor.

Repeated measures ANOVA was performed to assess responsiveness. The Mauchly test of sphericity was used, and when it was violated, technical corrections were performed using the Greenhouse-Geisser test. When the *F* test was significant, the Bonferroni test was used. Both floor and ceiling effects were evaluated descriptively through the 5^th^ and 95^th^ percentiles. Statistical significance was set at 5%. All analyses were performed using IBM® SPSS® 25.0 and R Project for Statistical Computing (lavaan package).

## Results

In total, 112 patients were evaluated for both reliability and construct validity of the Brazilian version of LoBACS. There were no missing data for these patients. The median age of the included patients was 48 (39.5–55.5) years, and the median duration of symptoms was 96 (36–210) months. Additional information about the study sample is presented in Table 1.

**Table 1:** Sample characteristics.

	n = 112
Gender _n (%)_	
Female	86 (76.8)
Male	26 (23.2)
Educational Level _n (%)_	
Incomplete Elementary School	13 (11.6)
Complete Elementary School	9 (8.03)
Incomplete High School	9 (8.03)
Complete High School	38 (33.9)
Incomplete Higher Education	11 (9.8)
Complete Higher Education	32 (28.6)
Age (years) _Md (25–75%)_	48 (39.5–55.5)
Body Weight (kg) _Md (25–75%)_	72 (63.75–83.3)
Height (m) _Md (25–75%)_	1.63 (1.58–1.70)
BMI (kg/m^2^) _Md (25–75%)_	27.34 (23.96–31.92)

6MWT = 6-Minute Walk Test; BMI = body mass index; Md = median; Kg = kilogram, and m = meters.

Regarding reliability, all the values were rated as excellent, except for the assessment of the intraobserver reliability of the Self-RegSE factor, which was considered good (Table 2).

**Table 2:** Inter- and intra-observer reliability results.

LoBACS-Brasil	ICC (95% CI)	SEM
Total		
*Inter-observer*	.90 (.84; .93)	-9.48
*Intra-observer*	.85 (.77; .91)	-7.51
FnSE		
*Inter-observer*	.93 (.89; .95)	-15.55
*Intra-observer*	.89 (.82; .93)	-13.04
Self-RegSE		
*Inter-observer*	.80 (.69; .87)	-6.94
*Intra-observer*	.63 (.42; .77)	- .42
ExSE		
*Inter-observer*	.87 (.79; .92)	-9.51
*Intra-observer*	.85 (.77; .91)	-8.64

ICC = Intraclass correlation coefficient; CI = confidence interval; SEM = standard error of the measurement; FnSE = functional self-efficacy; Self-RegSE = self-regulatory self-efficacy; ExSE = exercise self-efficacy.

**Fig 1:**
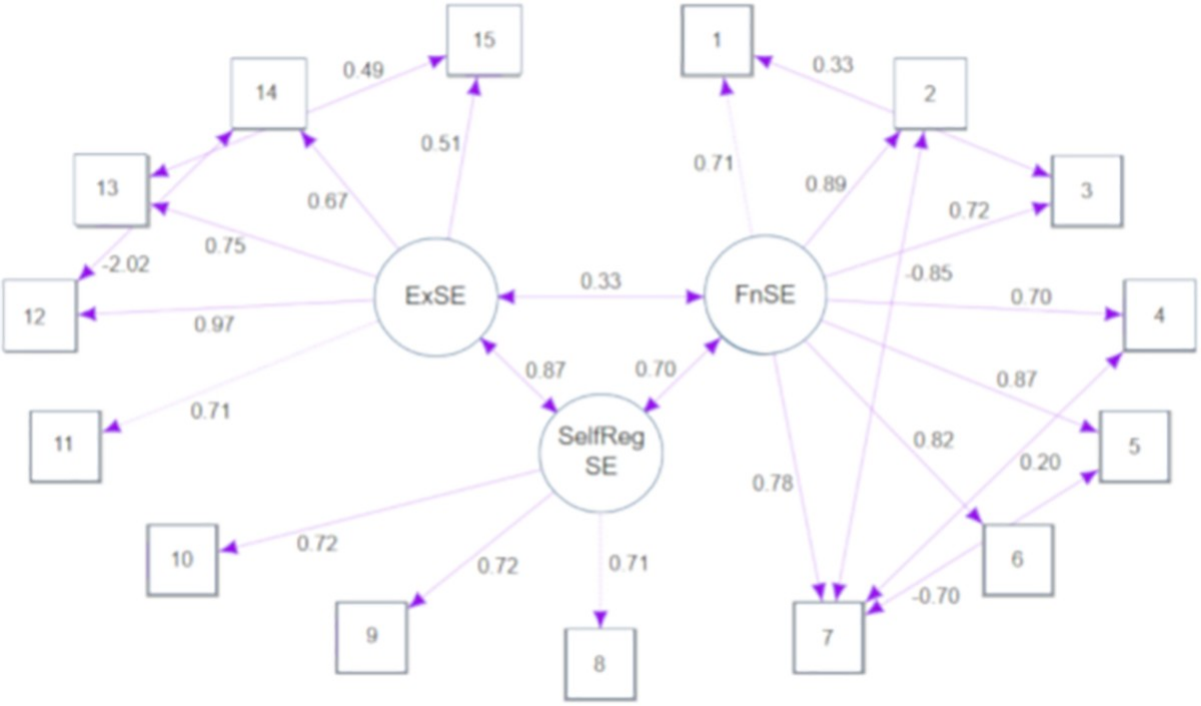
Path diagram.

The 3 factors found at the EFA were evaluated for their suitability using confirmatory factor analysis (CFA) [27]. Three models were set up to obtain a robust model (Fig 1). The results of the model fit tests were as follows: ᵪ^2^(81) = 179.79; *P*≤ .001; CFI = .95; TLI = .93 and root mean square error of approximation (RMSEA) = .10.

Regarding CR, the results were .85, .66, and .77 for FnSE, Self-RegSE, ExSE subscales, respectively. The Self-RegSE subscale presented problems of convergent validity, and the AVE value was .49. The AVE values for the FnSE and Self-RegSE subscales were .57 and .50, respectively. Subscales also presented divergent validity problems, with MSV values higher than AVE values for FnSE (.77), Self-RegSE (.77) and ExSE (.71). Regardless of this bias, LoBACS-Brasil presented satisfactory results in the comparison of standardized regression weights of the model, without versus with the common latent factor.

For the assessment of responsiveness, only 48 patients were evaluated because the remaining patients discontinued treatment for personal reasons. When the total score was evaluated, statistically significant differences were found in comparisons between the baseline evaluation (BE) and all subsequent applications (BE x 2^nd^ mean difference [MD] = −4.89; *P* = .03; BE x 4^th^ MD = −9.18; *P* < .001; BE x 6^th^ MD = −10.53; *P* < .001; BE x 8^th^ MD = −13.25; *P* < .001). The same pattern was found for the FnSE subscale. Unsatisfactory responsiveness patterns were found for the Self-RegSE and ExSE subscales. The 5^th^ and 95^th^ percentile values were, respectively, 38 and 95 for total score; 1 and 99 for FnSE; 24 and 100 for Self-RegSE, and 13 and 100 ExSE. These results indicate ceiling effects for both Self-RegSE and ExSE subscales.

## Discussion

The LoBACS-Brazil scale demonstrated excellent results for test–retest reliability, in line with the original study (FnSE, .92; Self-RegSE, .62; ExSE, .71; and total score, .85) [26]. The Self-RegSE subscale presented lower values of reliability in both the studies, and even lower values in the intrarater evaluation in our study. The inconsistent values can be explained by the subjective nature of the subscale and it being easily affected by the patient’s mood. Because data collection was performed on different days, patient mood could have varied. In addition, on the first evaluation day, the patients underwent few functional tests to provide timely treatment. This may have caused some pain to the patients, leading to the lower intrarater reliability results.

In our study the values in the model fit tests were acceptable. Furthermore, we found no bias in the database by the common latent factor, and the reliability as assessed by the composite reliability was adequate. However, subscales presented problems of both divergent and convergent validity, meaning that items did not correlate well with each other within their parent factor and that items correlated more highly with items outside their parent factor than with items within their parent factor. Hence, although the model indicates the existence of the three subscales, they are not independent of each other, thereby limiting their use.

Concerning responsiveness, we found statistical differences between comparisons of the BE and all other applications, for total score. These results indicate that the scale is responsive and that if the therapist applies the scale on the day of the BE, improvement of the patient’s self-efficacy throughout the treatment may be observed. The same pattern was observed for the FnSE subscale.

In contrast, the Self-RegSE and ExSE subscales did not present adequate standards of responsiveness. These findings can be justified by two reasons. First, the convergent and divergent validity of these subscales suggests that the LoBACS-Brasil should not be separated into subscales as it may lead to statistical problems. Second, the treatment to which the patients were submitted was not for self-efficacy and maybe that is why we did not find changes in the subscale scores. We could only observe improvements in aspects related to the treatment, specifically, the subscale related to functionality (FnSE) and total score. The problems of divergent and convergent validity may also explain the poor results related to ceiling effects of subscales.

Importantly, the original scale was validated in patients who recently received single-level lumbar microdiscectomy for the first time and the factorial analysis was performed in patients with post-acute LBP, which may result in differences found between the studies [26, 27].

Our study has some limitations. Although covariates such as the degree of pain and depression are known to influence self-efficacy [5, 26, 27, 33], they were not controlled in this study. Personal self-efficacy develops from sources such as previous experiences, observations, verbal/society persuasion, and physiological state [34] and is mediated by the interpretation of specific events [35]. Therefore, this cognitive factor is highly important and demonstrates how situations such as depression can influence self-efficacy [35]. In addition, the questionnaires were applied through interview because of the educational condition of the study sample, whereas the original version was self-administered. This difference could have influenced the results.

### Implications for research

Further studies must control covariates such as pain and depression to clarify their effects on the LoBACS-Brasil score. The validity of the scale must be evaluated using a more specific tool. Prospective studies with a specific objective of self-efficacy improvement are required to provide better insights into responsiveness and to establish cutoff points for the scale.

### Implications for practice

Self-efficacy is an important outcome to be evaluated in patients with CLBP, especially when considering the biopsychosocial model of the disease. Validation of LoBACS in Brazilian–Portuguese presents the professional with the possibility of a differentiated assessment of the patient, provides information on confidence in performing functional activities and coping strategy adopted by the patient, and predicts their possible adherence to exercise treatment. In addition, the use of objective and reliable tools assist in thorough evaluation, evidence-based prescription, and a systematic strategy to monitor the clinical course.

## Conclusions

Only the total score of the Brazilian version of LoBACS proved to be reliable, valid, and responsive for use in patients with CLBP. Although factorial analyses indicate the existence of three subscales, these should not be used due to problems associated with convergent and divergent validity, responsiveness, and ceiling effects.

## Supporting information

S1 File Low Back Activity Confidence Scale–LoBACS-Brasil. (PDF)

S2 File LoBACS-Brasil_Descriptive database. (SAV)

S3 File LoBACS-Brasil_Factorial database. (SAV)

S4 File LoBACS-Brasil_ Responsiveness database. (SAV)

S1 Table Aquatic and Deep-Water Running Exercises Program. (PDF)
