# Supplementary material for: Assessment of measurement properties of the Brazilian-Portuguese version of the low back activity confidence scale (LoBACS) in patients with chronic low back pain
Source: PLoS One. 2020 Sep 22;15(9):e0239332. doi: 10.1371/journal.pone.0239332 (PMC7508363; doi:10.1371/journal.pone.0239332)
Supplement: S1 File — (PDF) [file pone.0239332.s001.pdf]

Nome: ____**Data**:___/___/___
Data de Nascimento: ___/___/___ **Gênero:** ( )Masc ( )Fem

Low Back Activity Confidence Scale – LoBACS Brasil

Os seguintes itens tem o objetivo de determinar quais tipos de atividades você pode fazer com facilidade, quais são mais difíceis e quais você não consegue fazer com sucesso. **Por favor, indique seu grau de confiança, neste presente momento, para fazer a atividade em questão, circulando o número apropriado.** Selecione a resposta que mais se aproxima com você, lembrando que não há respostas certas ou erradas.

Por exemplo, no item 1 se você tem quase confiança completa de que você poderia mover um livro pesado de uma estante acima da sua cabeça para uma estante mais baixa, você pode **circular 90%.** Se, entretanto, você não tiver confiança alguma que você poderia movê-la, você **circularia 0%.**

0% 10% 20% 30% 40% 50% 60% 70% 80% 90% 100%

Nenhuma confiança **Confiança moderada** **Confiança total**

**Eu acredito que eu posso...**											
	%	%	%	%	%	%	%	%	%	%	%
1. Carregar um objeto de 10 quilos do carro para dentro da minha casa.	0	10	20	30	40	50	60	70	80	90	100
2. Mover um livro pesado de uma estante acima da minha cabeça para uma estante mais baixa.	0	10	20	30	40	50	60	70	80	90	100
3. Empurrar um sofá grande por 3 metros para dentro de outro local sobre uma superfície áspera (por exemplo, um carpete).	0	10	20	30	40	50	60	70	80	90	100
4. Permanecer sentado por 6 horas em uma viagem.	0	10	20	30	40	50	60	70	80	90	100
5. Subir 3 lances de escada (30 degraus).	0	10	20	30	40	50	60	70	80	90	100
6. Caminhar 1,5 quilômetros (15 quadras/quarteirões de uma cidade) sem parar.	0	10	20	30	40	50	60	70	80	90	100
7. Ficar em pé em uma fila lenta por 3 horas.	0	10	20	30	40	50	60	70	80	90	100
8. Seguir as recomendações para cuidar das minhas costas.	0	10	20	30	40	50	60	70	80	90	100
9. Controlar meu problema da coluna lombar de maneira que eu consiga fazer coisas de que eu gosto.	0	10	20	30	40	50	60	70	80	90	100
10. Encontrar forças dentro de mim para lidar com a frustação da dor em minha coluna.	0	10	20	30	40	50	60	70	80	90	100
11. Continuar a fazer meus exercícios, mesmo quando eu tenho dor ou desconforto.	0	10	20	30	40	50	60	70	80	90	100
12. Continuar a fazer meus exercícios mesmo se eu não tenho sintomas atuais do meu problema na coluna.	0	10	20	30	40	50	60	70	80	90	100
13. Exercitar-me regularmente mesmo se eu estiver chateado (a) com o programa ou a atividade.	0	10	20	30	40	50	60	70	80	90	100
14. Exercitar-me quando não há alguém ao redor para oferecer encorajamento.	0	10	20	30	40	50	60	70	80	90	100
15. Voltar a realizar meu programa de exercícios após tê-lo abandonado.	0	10	20	30	40	50	60	70	80	90	100

Pontuação:
Média dos itens 1-7: ____________Média dos itens 8-10: ____________ Média dos itens 11-15: ____________Média dos itens 1-15: ____________
