# Supplementary material for: Assessment of measurement properties of the Brazilian-Portuguese version of the low back activity confidence scale (LoBACS) in patients with chronic low back pain
Source: PLoS One. 2020 Sep 22;15(9):e0239332. doi: 10.1371/journal.pone.0239332 (PMC7508363; doi:10.1371/journal.pone.0239332)
Supplement: S1 Table — (PDF) [file pone.0239332.s005.pdf]

S2 Table. Aquatic and Deep-Water Running Exercises Programme.

Week	Description of Exercises	Repeats × Time
1st–4th<br>5th–8th	Walking forwards, sideways and backwards.<br>Walking forwards, sideways and backwards. With palm and leggings with elastic resistance.	1 × 10 min<br>1 × 10 min
1st–8th	Active pelvic mobilisation (anteversion, retroversion and laterolateral), standing with slightly bent knees, supports the back on the edge of the pool.	2 × 30 s
1st–4th<br>5th– 8th	Lumbar spine stabilisation: isometric trunk muscles with legs together, knees and hip flexion, resisting move with the turbulence made by the physiotherapist. Bipodal.<br>The same: unipodal.	1 × 60 s<br>1 × 30 s per leg
1st–4th<br>5th–8th	Dynamic exercises (flexion and extension, abduction and adduction) for the hip joint muscles, the patient with one hand fixed in the pool bar and the other on her hip. Keeping your body upright with axial growth and isometric muscle of the abdomen and lumbar spine.<br>The same: with additional intensity through leggings with elastic resistance.	1 × 60 s per each exercise<br>1 × 60 s per each exercise
1st–8th	Transversal rotation with 'spaghetti' float: the patient performs the rotational movement forwards and backwards without setting foot on the floor and minimal physical therapist support. Adapting the exercise of the Halliwick Method.	1 × 60 s
1st–4th<br>5th–8th	Bridge Bipedal: patient supine with float neck brace and 'spaghetti' float below the feet and performs hip extension movement with knee flexion and the physiotherapist supports with one hand on the patient's sacrum. Bipodal.<br>The same: unipodal.	2 × 60 s<br>1 × 60 s per leg
5th–8th	Method Bad Ragaz: trunk lateral flexion (patient supine with float neck brace and fixed physiotherapist lower limbs between the elbow and trunk and hands fixed on the hip of the patient), trunk flexion (patient supine with float neck brace and physiotherapist fixing the lower limb between the elbow and trunk and hands fixed in trochanter of the femur of the patient to perform the exercise of abdominal), trunk extension (patient supine with float neck brace and fixed physiotherapist lower limbs between the elbow and trunk and hands fixed in the hip of the patient, the patient performed trunk extension movement and the physiotherapist rotates the longitudinal axis of the patient to one side and sliding it back as the patient performs the extension.	1 × 60 s per each exercise
1st–8th	Lumbar Traction: patient supine can be fixed with the hands in the pool bar and the physiotherapist performs traction, an alternative is to carry out this exercise in Watsu position.	2 × 30 s

1st–4th<br><br><br><br><br><br>5th–8th	Axial traction in the position that the patient is abreast the edge of the pool and fixed hands on the bar, flexes the hip with knee extension, standing and physical therapist places a hand on the base of the occipital and the other at the base of the sacrum performs traction with expiration into the water.<br>The same: dorsiflexion.	2 × 30 s<br><br><br><br><br><br>2 × 30 s
1st–8th	Neural mobilisation of the posterior chain: stretching of the posterior muscles, standing, leaning back on the edge of the pool, the physiotherapist stands on one side and puts a hand on the base of the occipital and the other ankle. Raises the lower limb (hip flexion) with knee extension and flexes the head and trunk.	2 × 30 per side
1st–8th	Specific stretching exercises for the muscles: quadratus lumborum, piriform and iliopsoas.	2 × 30 s
1st–2nd<br>3rd–8th	Deep-Water Running with RPE around 11.<br>Deep-Water Running with RPE around 15.<br>Cool down.	20 min<br>20 min<br>3–5 min
1st–8th	Relaxation: the physiotherapist performs Watsu or massage of the low back with ball massager.	5 min

Ref: Carvalho RGS, Silva MF, Dias JM, Olkoski MM, Dela Bela LF, Pelegrinelli ARM *et al*. Musculoskel Sci Prac. 2020; 49:102195.
